# Predicting MoRFs in protein sequences using HMM profiles

**DOI:** 10.1186/s12859-016-1375-0

**Published:** 2016-12-22

**Authors:** Ronesh Sharma, Shiu Kumar, Tatsuhiko Tsunoda, Ashwini Patil, Alok Sharma

**Affiliations:** 1 School of Electrical and Electronics Engineering, Fiji National University, Suva, Fiji; 2 School of Engineering and Physics, The University of the South Pacific, Suva, Fiji; 3 CREST, JST, Yokohama, 230-0045 Japan; 4 RIKEN Center for Integrative Medical Science, Yokohama, 230-0045 Japan; 5 Medical Research Institute, Tokyo Medical and Dental University, Tokyo, 113-8510 Japan; 6 Human Genome Center, The Institute of Medical Science, The University of Tokyo, Tokyo, Japan

**Keywords:** Molecular recognition features, Hidden Markov model profiles, Intrinsically disordered proteins, Intrinsically disordered regions, Support vector machines

## Abstract

**Background:**

Intrinsically Disordered Proteins (IDPs) lack an ordered three-dimensional structure and are enriched in various biological processes. The Molecular Recognition Features (MoRFs) are functional regions within IDPs that undergo a disorder-to-order transition on binding to a partner protein. Identifying MoRFs in IDPs using computational methods is a challenging task.

**Methods:**

In this study, we introduce hidden Markov model (HMM) profiles to accurately identify the location of MoRFs in disordered protein sequences. Using windowing technique, HMM profiles are utilised to extract features from protein sequences and support vector machines (SVM) are used to calculate a propensity score for each residue. Two different SVM kernels with high noise tolerance are evaluated with a varying window size and the scores of the SVM models are combined to generate the final propensity score to predict MoRF residues. The SVM models are designed to extract maximal information between MoRF residues, its neighboring regions (Flanks) and the remainder of the sequence (Others).

**Results:**

To evaluate the proposed method, its performance was compared to that of other MoRF predictors; MoRFpred and ANCHOR. The results show that the proposed method outperforms these two predictors.

**Conclusions:**

Using HMM profile as a source of feature extraction, the proposed method indicates improvement in predicting MoRFs in disordered protein sequences.

## Background

The role of Intrinsically Disordered Regions (IDRs) in protein function has been well studied [1]. IDRs lack a fixed three-dimensional structure under physiological conditions and can adopt an ensemble of conformations. They are associated with important cellular processes, such as signal transduction and transcriptional regulation [2, 3]. MoRFs are short binding regions of length 5 to 25 residues present within longer disordered protein sequences [4, 5]. They undergo a disorder-to-order transition on binding their partner proteins. Upon binding, they can adopt various conformations including α-helix (*α*-MoRFs), *β*-strand (*β*-MoRFs), *γ*-coil (*γ*-MoRFs) or mixtures of these (complex-MoRFs) [5].

Identifying the binding regions in IDPs is a challenging task in bioinformatics and a growing area of interest [6]. Pattern recognition approaches involving the development of feature extraction techniques and classifiers have been used to locate binding regions in IDPs. To develop computational approaches to identify the binding regions, recently two main approaches have been used in the literature [4, 7, 8]. The first approach is based on the identification of short linear motifs (SLiMs) which are conserved sequences of size 3 to 10 amino acids [7]. On the other hand, the second approach addresses long interaction segments present in IDPs called MoRFs, which are also conserved but vary in size, can be up to 70 amino acids and are often described as disordered domains.

Several predictors have been developed to identify SLiMs and MoRFs in disordered protein sequences [7, 8], namely, MoRFpred [8], ANCHOR [9, 10], MFSPSSMpred [11], γ-MoRF-PredII [12], SliMpred [13], SLiMDis [14] and SliMFinder [15]. Considering all of the above predictors, the methods for identifying SLiMs and MoRFs are different even though SLiMs and MoRFs interact within IDRs. With the short lengths of SLiMs, the prediction of SLiMs in the IDR sequence is very challenging and their identification has a high false positive rate (FPR). On the other hand, predicting MoRFs from IDR sequences is comparatively easier due to their greater average length. The overlapping of SLiMs and MoRFs make the prediction scheme more challenging, however in this work we only focus on the identification of MoRFs from computational perspective as previously outlined in Disfani et al. [8].

Most of the available disorder predictors have been benchmarked by comparing their performance to those of MoRFpred and ANCHOR which have very different prediction approaches. ANCHOR is a downloadable predictor and uses properties of residues in the protein sequence to predict MoRFs [10]. These properties are as follows: the binding regions must be present in a long disordered region, query residues do not fold with neighboring residues and do not interact with global domains. Using each of these properties for prediction, a propensity score is generated by utilising energy estimation approach of IUPred (IDR predictor) [16] and a weighted sum is used to produce the final propensity score. On the other hand, MoRFpred [8] is a web-based predictor and utilizes nine sets of features to generate a propensity score for a residue. These features are extracted from the physicochemical properties of residues within the protein sequence, position specific scoring matrices (PSSM) extracted using PSI-BLAST [17], relative solvent accessibility given by Real-SPINE3 [18], flexibility (B-factor) estimated by PROFbval [19], and the predictions of five different intrinsic disorder predictors are used. Finally, using PSI-BLAST [17], MoRFpred aligns the query sequence to the training sequences and calculates an e-value for the prediction.

We propose a new approach of utilising evolutionary information for identifying MoRFs in IDR sequences. First, the input protein sequence is transformed into a feature vector that represents the discrimination information between MoRF regions and the surrounding IDRs. Next, the feature vectors are fed to a SVM model to generate propensity scores for the residues. Our approach involves two novel aspects which makes the proposed method a good predictive scheme. First, we extract sequence features encoded in HMM profiles, which has not been previously explored for MoRF prediction. Second, we use a unique architecture that selects and combines appropriate SVM models to generate the final propensity scores for the residues. Moreover, using only HMM profiles, our approach is more accurate than ANCHOR and MoRFpred. ANCHOR and MoRFpred achieved AUC values of 0.600 and 0.673, respectively, whereas the proposed method achieves higher AUC value of 0.70.

## Methods

### Benchmark dataset

We used the data set that was previously used to benchmark MoRFpred [8] and ANCHOR [10] predictors. To create this dataset, Disfani et al. [8] used structures of protein-peptide interactions from Protein Data Bank (PDB) [8, 20, 21]. Structures with peptide regions of 5 to 25 residues were selected and assumed to be a MoRF region. This resulted in 840 protein sequences. To develop and analyse MoRF predictors, Disfani et al. [8] divided these 840 protein sequences into 421 train sequences and 419 test sequences. The training set contains 5,396 MoRF residues and 240,588 non-MoRF residues, whereas the test set contains 5,153 MoRF residues and 253, 676 non-MoRF residues.

### Overview of the proposed method

Computationally identifying MoRF residues in disordered protein sequences requires the process of developing feature extraction techniques and classifiers. Using feature extraction technique, important features are extracted to represent protein sequence region and in classification task, these features are used to predict the location of MoRF residues in the disordered region. Features representing a MoRF from the protein sequence can be extracted in a number of ways using syntactical and physicochemical properties [22, 23], structural information [24] and using evolutionary information [24–27]. Early studies focused on the use of syntactical, physicochemical properties and structural information of protein sequences. Recently, the use of evolutionary information from protein sequences has resulted in better prediction accuracies [26, 28, 29].

To extract evolutionary features, either PSI-BLAST can be used to generate position specific scoring matrix (PSSM) or HHblits can be used to generate hidden Markov model (HMM) profile. Both PSSM and HMM are sequence profiles. To generate evolutionary profiles, PSI-BLAST or HHblits searches a protein sequence database, finds similar protein sequences and computes sequence profiles that contain the substitution probability of each amino acid based on its position. According to previous studies [26, 29, 30], HHblits is faster and more accurate compared to PSI-BLAST. In this study, features are extracted from HMM profiles and an SVM classifier is used for the prediction of MoRF residues in protein sequences.

**Fig. 1:**
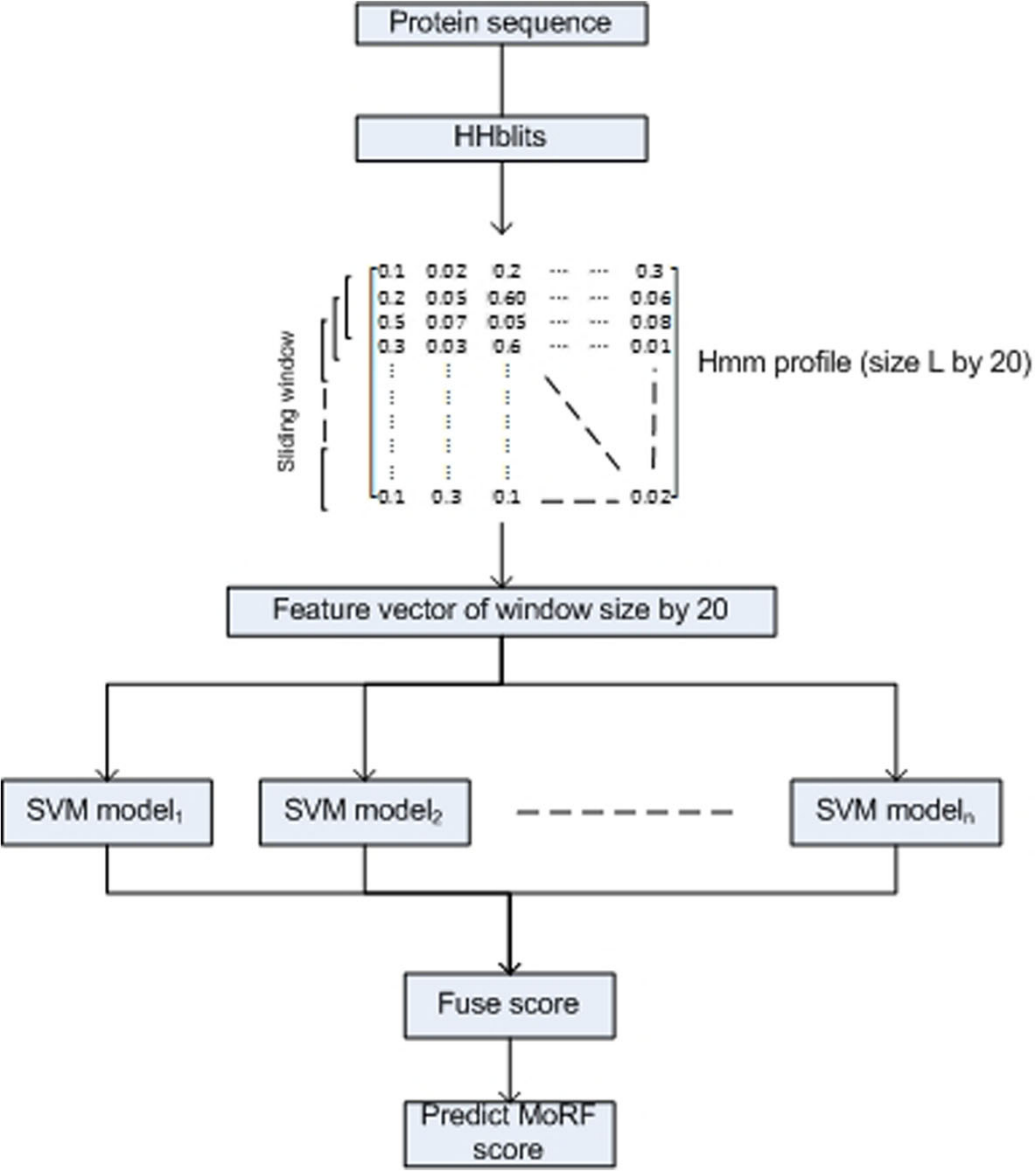
Overview of the proposed method

Figure 1 shows the overview of the proposed method. The proposed method first computes HMM profiles using HHblits. Using these HMM profiles, feature vectors are extracted using a sliding window to represent each residue in the input query protein sequence. A feature vector of size w × 20 (where w is the window size and number 20 represents the selected number of columns of HMM profile) is given to a LibSVM [31] classifier to compute the propensity of a query residue being a part of a MoRF. Finally, different SVM models are processed to fuse the propensity score of the residues to be predicted.

### HMM profiles

HMM profiles are computed using HHblits [30]. HHblits iteratively searches through databases and finds significantly similar sequences to build high quality multiple sequence alignments (MSAs) either from single sequence or MSAs itself [30]. To represent MSAs more concisely, after each iterative search HHblits transforms the MSAs into query HMM profiles. These HMM profiles contain 20 common amino acids in homologous proteins and for each amino acid a substitution probability is provided based on its position along the length of the protein sequence. Compared with other sequence profiles, HMM profiles contain 10 additional columns which represent the probabilities observing insertion, deletion and match during MSAs.

Using NR20 protein database and setting cut off value (E) of HHblits to 0.001, HMM profiles are computed for each protein sequence in four iterations. For a given protein sequence of length *L*, the HHblits outputs HMM profile matrix of size $L\times 30$. The values in HMM profile are transformed to linear probabilities using the equation $p={2}^{-N/1000}$, where $N$ is the score number from the profile. For evaluation of the benchmark used in this study, we only use first 20 columns of HMM profile.

### Training

**Fig. 2:**
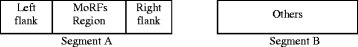
Two segments from each training sequences discriminating MoRFs region from other surroundings of IDR

In the initial stage, positive and negative samples of the training dataset are defined. As in Disfani et al., each sequence is divided into three regions (MoRFs, Flanks and Others). MoRF regions are annotated with known MoRFs, Flanks (12 amino acid to the right of MoRF and 12 amino acid to the left of MoRF) and remaining amino acids are denoted as Others. For training, if the length of the Flank regions is less than 12 amino acids due to MoRFs being present at the start or end of a sequence, zeros are inserted in the Flank region. To generate features for training, two segments (segment A and segment B) are developed for each sequence using the three regions as shown in Fig. 2.

Balanced sampling was enabled by extracting positive samples from segment A and randomly selecting the same number of negative samples from segment B. For each of the MoRF residues present in segment A, windowing technique is utilised and MoRF residue information, right neighbor region information (maximum of 12 amino acids) and left neighbor region information (maximum of 12 amino acids) are taken. Thus, features are generated from a segment centered on the input residue which is to be predicted. The number of positive samples for each sequence is equal to the number of MoRF residues per each sequence. To avoid over fitting in the process of training, non-MoRF residues that are not part of the Flanks of MoRF regions are selected. This is followed by random selection of same numbers of negative samples from segment B using the above procedure. The number of negative samples are increased to ratio 1:2, this gives twice as many negative samples compared to positive samples (2 non-MoRF residue segments for each MoRF residue segment). This ratio is also increased to 1:3 (3 non-MoRF residue segments for each MoRF residue segment) and the best ratio for training is selected by comparing the performance matrices. Furthermore, to guarantee unbiased prediction different sets of non-MoRF residue segments are randomly selected for each model with different window size.

### Testing

To score a query protein sequence, the proposed method uses a sliding window to compute features. Since the size of the MoRF is not known, the center of the window is placed on the query residue to be predicted and the Flank size is varied on both sides for evaluation. For each varying window, the features are computed and processed using the SVM classifier.

### SVM model and score fusion

We used two different SVM kernels, radial basis function (RBF) and sigmoid to evaluate the evolutionary information. Using each of the SVM kernels with window size of 7 in the windowing technique (w was selected as 7 due to the processing time), C and gamma values with best AUC, success rate and FPR were selected and used to evaluate the evolutionary information by varying the window size. Finally, best performing SVM models are selected and common averaging is applied to fuse the output score of each model. In common averaging output scores of all selected models are added and the sum is divided by the total number of models used.

### Performance measure

To appropriately rank and compare the proposed method with the available MoRF predictors, we used three evaluation metrics. These are AUC (area under the ROC curve), success rate and accuracy. These evaluation metrics have been previously used to compare and analyse MoRF predictors [8, 20, 21] and are described in detail by Disfani et al. [8]. Success rate is used to analyse and compare the mean predicted propensity scores of real MoRF residues to that of non-MoRF residues. Accuracy is defined as the percent of residues that are correctly classified as MoRFs and non-MoRFs.

## Results

Appropriate SVM models with selected features were identified for the proposed method and the proposed method was evaluated using a test set. The performance matrices are compared with MoRFpred and ANCHOR predictors.

### SVM model and feature selection

The dataset used in this study has more non-MoRF residues compared to the number of MoRF residues present in the sequences resulting in a biased prediction. To overcome this, three approaches are taken to under sample non-MoRFs residues during training, parameterization and feature selection. First, non-MoRF residues that do not interact with Flanks of the MoRFs region are selected. Second, random sampling is used to select two non-MoRF residues for each MoRF residue (2:1 ratio between non-MoRFs and MoRFs residue). The ratio is also extended to 3:1 using the entire surrounding of the MoRF and Flank regions within the IDR sequence to select non-MoRF residues. Moreover, each time, different sets of non-MoRF residue segments are randomly selected for each model.

Features and SVM models are selected using three criteria: empowering high AUC, high success rate and lower FPR. To achieve these goals, the SVM models are parameterized and the window size w in the windowing technique is varied in order to extract appropriate features from HMM profiles. Next, each set of selected features are fed to the SVM model with different kernels and gamma values. Performing grid search, C value of 1000 was approximated for both kernels producing best AUC, success rate and FPR, while gamma value of 0.0038 was selected for RBF kernel and gamma value of 5 was selected for sigmoid kernel to produce best AUC and FPR. Moreover gamma value of 5 was also selected for RBF kernel to produce high success rate. Finally three sets of SVM models (RBF kernel: C = 1000, gamma = 0.038; RBF kernel: C = 1000, gamma = 5; Sigmoid kernel: C = 1000, gamma = 5) were selected to evaluate each set of features generated by varying the window size.

Table 1 summarizes the results for feature and model selection. FPR is computed as a function of TPR. We used TPR value of 0.222, first, to directly compare the proposed method with ANCHOR and MoRFpred predictors and second, TPR near a lower value of FPR produces higher propensity scores for real MoRF residues. Considering average values of AUC, success rate and FPR, the best nine performing models are selected and their scores are fused to generate the final propensity score for each residue. Table 2 outlines the selected models. For each of the selected models, the sampling ratio is increased to 1:2 and 1:3 between MoRF residue segment and non-MoRF residue segment during training. Table 3 shows the three performance matrices with increasing sampling ratio from 1:1 to 1:2. Increasing sampling ratio to 1:3 did not work out well and gave over prediction results. The best performing model were selected from Table 3. As expected the models individually over predict MoRFs as observed in Table 1; they have comparatively high FPR and moderately low success rates and AUCs. Therefore, it can be concluded that these models could not correctly identify MoRFs alone. Selecting best performing models and fusing their scores using common averaging, we are able to achieve good AUC, success rate and FPR as observed in Tables 4 and 5.

**Table 1:** AUC, Success rate and FPR for varying flank size with RBF and sigmoid kernels (C value used is 1000)

	AUC				Success rate				FPR @ 0.222 TPR			
	RBF kernel		Sigmoid kernel		RBF kernel		Sigmoid kernel		RBF kernel		Sigmoid kernel	
Gamma	0.0038	5	0.0038	5	0.0038	5	0.0038	5	0.0038	5	0.0038	5
Flank size												
1	0.658	0.587	0.570	**0.648**	0.680	0.660	0.658	**0.701**	0.057	0.160	0.090	**0.070**
2	0.659	0.597	0.590	**0.653**	0.651	0.737	0.653	**0.680**	0.053	0.190	0.088	**0.065**
3	**0.650**	**0.600**	0.580	**0.650**	**0.640**	**0.770**	0.660	**0.660**	**0.047**	**0.180**	0.080	**0.065**
4	0.660	**0.606**	0.580	0.340	0.640	**0.770**	0.589	0.370	0.053	**0.180**	0.090	0.380
5	**0.660**	0.600	0.587	0.650	**0.669**	0.720	0.618	0.600	**0.050**	0.180	0.098	0.060
6	**0.659**	**0.600**	0.589	0.648	**0.649**	**0.749**	0.618	0.572	**0.053**	**0.180**	0.098	0.066
7	0.664	0.601	0.588	0.340	0.644	0.756	0.642	0.460	0.051	0.170	0.090	0.360
8	0.652	0.602	0.595	0.350	0.653	0.740	0.600	0.470	0.059	0.170	0.095	0.360
9	0.646	0.584	0.582	0.618	0.653	0.699	0.584	0.390	0.061	0.180	0.010	0.073
10	0.644	0.587	0.640	0.590	0.656	0.699	0.432	0.590	0.065	0.175	0.077	0.100
11	0.645	0.605	0.640	0.639	0.668	0.749	0.604	0.390	0.066	0.160	0.105	0.080
12	0.640	0.600	0.600	0.630	0.670	0.810	0.630	0..36	0.070	0.160	0.090	0.080

Bold numbers indicate the best performance metrics for different kernels, gamma values and Flank sizes

**Table 2:** Selected SVM models with respective gamma and window size values

SVM models	window size	kernel	gamma
1	11	RBF	0.0038
2	7	RBF	5
3	3	Sigmoid	5
4	13	RBF	0.0038
5	9	RBF	5
6	5	Sigmoid	5
7	7	RBF	0.0038
8	13	RBF	5
9	7	Sigmoid	5

**Table 3:** Selected SVM models with increased sampling ratio

Training sampling ratio	1:1			1:2		
SVM models	AUC	Success rate	FPR	AUC	Success rate	FPR
1	0.660	0.669	0.050	**0.680**	**0.637**	**0.041**
2	0.600	0.770	0.180	**0.613**	**0.730**	**0.175**
3	0.648	0.701	0.070	**0.650**	**0.690**	**0.070**
4	0.659	0.649	0.053	**0.680**	**0.620**	**0.042**
5	**0.606**	**0.770**	**0.180**	0.600	0.700	0.190
6	0.653	0.680	0.065	**0.654**	**0.680**	**0.063**
7	0.650	0.640	0.047	**0.660**	**0.640**	**0.045**
8	0.600	0.749	0.180	**0.610**	**0.726**	**0.175**
9	**0.650**	**0.660**	**0.065**	0.650	0.656	0.065

Bold numbers indicate performance metrics for best models

**Table 4:** Comparison of results

Method/predictors	TPR	AUC	Success rate	FPR	Accuracy
ANCHOR	0.222	0.600	0.611	0.894	0.092
MoRFPred	0.222	0.673	0.718	0.037	0.948
Proposed method	0.222	0.702	0.711	0.036	0.949

Accuracy and FPR is a function of TPR and the underlined values are obtained from Disfani et al. [8]

**Table 5:** Overall Comparison of results

	Proposed method	MoRFPred	ANCHOR
Efficiency residues/min	405	48	4 × 10^6^
Max sequence size	Unlimited	1000 residues	Unlimited
AUC	0.702	0.673	0.600
FPR at 0.222 TPR	0.036	0.037	0.092
FPR at 0.389 TPR	0.109	0.137	0.253
Number of component predictors	1	8	0
MoRF size limitations	No limits	No limits	No limits

### Comparison with MoRFpred and ANCHOR predictors

The proposed method is empirically compared with predictors MoRFpred and ANCHOR. Table 4 shows the AUC, success rate, and FPR of the two predictors together with that of the proposed method. From the comparison, it is noted that the proposed method achieves relatively higher AUC value when compared with AUC obtained from ANCHOR and MoRFpred. This is a clear indication that the proposed method outperforms the two mentioned predictors in terms of success rate, FPR and accuracy. Even though our method utilizes only one component predictor compared to 8 component predictor used by MoRFpred, our method achieves higher AUC and best FPR.

## Discussion

A novel approach of using evolutionary information for the prediction of MoRFs in disordered protein sequences is proposed. The performance of the proposed method is compared with ANCHOR and MoRFpred. The results clearly demonstrate that the proposed method outperforms the two predictors in terms of AUC, accuracy and FPR. Since MoRF predictors are used to score large number of protein sequences, they need to be analysed in terms of their efficiency. We tested our proposed method and ANCHOR using Intel core i5 3.5G desktop, whereas MoRFpred was tested by submitting input sequence to the webserver. In terms of processing speed, MoRFpred is slowest at 48 r/m (residues/min), ANCHOR is fastest at 4 × 10^6^ r/m and our method came at 405 r/m. Though the processor speed for the MoRFpred web server is not known, comparing AUC, accuracy and FPR of these predictors, our proposed method offers a good performance at a reasonable processing speed. Prediction time for ANCHOR is fastest, since it does not rely on PSI-BLAST, whereas MoRFpred relies on PSI-BLAST and is slowest in predicting MoRF in protein sequences.

The proposed method relies on HHblits, which computes evolutionary profiles at a higher processing speed compared PSI-BLAST. The use of HHblits in the proposed method offered much higher predicting speed compared to MoRFpred. Though ANCHOR is the fastest method, the proposed method is more accurate.

Overall, we have proposed a new sequence profile based MoRF predictor, which offers promising performance and processing speed compared to ANCHOR and MoRFpred predictors, respectively. The success behind the proposed predictor relies on the use of a large training dataset, use of HMM profiles derived from fast and accurate MSAs and the unique architecture that combines different SVM-based models.

The use of evolutionary information (HMM profiles) provides a comprehensive set of features to distinguish the properties of predicted residues along its Flank region in the sequence resulting in performance improvement of the proposed method. In general, to predict MoRF scores, one would want the MoRF predictor to be consistent over the entire query sequence. However, if the MoRFs in the query sequence are very similar to the training samples, these MoRFs will be scored more positively compared to other MoRFs in the query sequence. This would result in a biased prediction and could obstruct the identification of novel MoRFs. Different learning methods show different biases with similar training datasets. For example, SVM classifiers with a RBF kernel tend to over score their training data, while those with a sigmoid kernel tend to avoid over scoring, as observed in Table 1 for each models with two different kernels.

The proposed method utilizes several approaches during training to avoid over prediction or under prediction. These are, the use of RBF and sigmoid kernels, the use of non-MoRF residues that are not part of the Flanks of MoRF regions, selecting suitable ratios between MoRF and non-MoRF residue samples and finally randomly selecting non-MoRF residue samples for each model. Using common averaging to fuse propensity scores generated by different models using different sets of features makes the proposed method less susceptible to make a biased prediction when compared to single model prediction.

The proposed predictor was compared with available predictors, ANCHOR and MoRFPred. While these methods provide a propensity score and a binary prediction value for each residue, the proposed method only includes a numerical propensity score value since different protein sequence might have different levels of predicted propensity thresholds.

Further, ANCHOR is downloadable and fast but is limited in prediction accuracy, whereas, MoRFpred is provided as a web based predictor allowing limited input and cannot be used for a large number of query sequences. The proposed predictor is available in the form of MATLAB code and uses HMM profiles for prediction of MoRFs. It is fast, accurate and without any limitation when compared with ANCHOR and MoRFpred. This makes the proposed predictor useful in the analysis of other datasets and it can also be used as an input component to other application.

The MATLAB codes, train and test sets and the documentation for the proposed method are available at the web-link:

https://github.com/roneshsharma/Predict-MoRFs

## Conclusions

In this study, HMM profiles for identifying MoRF residues in protein sequence have been used. The comparison of the performance parameters clearly demonstrate that the proposed method outperforms ANCHOR and MoRFpred predictors.

## References

[1] Tompa P (2011). Unstructural biology coming of age. Curr Opin Struct Biol..

[2] Dyson HJ, and Wright PE. Intrinsically unstructured proteins and their functions. Nat Rev Mol Cell Biol. 2005;6:197-208. 10.1038/nrm1589 15738986

[3] Tompa P (2005). The interplay between structure and function in intrinsically unstructured proteins. FEBS Lett.

[4] Das RK, Mao AH, Pappu RV. Unmasking functional motifs within disordered regions of proteins.Bioinformatics. 2012;5:pe17. doi: 10.1126/scisignal.2003091. 10.1126/scisignal.2003091 22510467

[5] Mohan A, Oldfield CJ, Radivojac P, Vacic V, Cortese MS, Dunker AK, Uversky VN (2006). Analysis of molecular recognition features (MoRFs). Mol Biol.

[6] Huang J, and Li S. Mining p53 binding sites using profile hidden Markov model, Proceedings of the International Conference on Information Technology: Coding and Computing (ITCC’05). 2005;1:146–151

[7] Davey NE, Cowan JL, CShields D, Gibson TJ, Coldwell MJ, Edwards RJ (2012). SLiMPrints: conservation-based discovery of functional motif fingerprints in intrinsically disordered protein regions. Nucleic Acids Res.

[8] Disfani FM, Hsu WL, Mizianty MJ, Oldfield CJ, Xue B, Dunker AK, Uversky VN, Kurgan L (2012). MoRFpred, a computational tool for sequence-based prediction and characterization of short disorder-to-order transitioning binding regions in proteins. Bioinformatics.

[9] Mészáros B, Simon I, and Dosztányi Z. Prediction of protein binding regions in disordered proteins. PLoS Comput Biol. 2009; 5:e1000376. doi: 10.1371/journal.pcbi.1000376. 10.1371/journal.pcbi.1000376 PMC2671142 19412530

[10] Dosztányi Z, Mészáros B, Simon I (2009). ANCHOR: web server for predicting protein binding regions in disordered proteins. Bioinformatics.

[11] Fang C, Noguchi T, Tominaga D, and Yamana H. MFSPSSMpred: identifying short disorder-to-orderbinding regions in disordered proteins based on contextual local evolutionary conservation. BMC Bioinformatics. 2013;14:300. doi: 10.1186/1471-2105-14-300. pmid:24093637. 10.1186/1471-2105-14-300 PMC3853019 24093637

[12] Cheng Y, Oldfield CJ, Meng J, Romero P, Uversky VN, Dunker AK (2007). Mining alpha-helix-forming molecular recognition features with cross species sequence alignments. Biochemistry.

[13] Mooney C, Pollastri G, Shields DC, Haslam NJ (2012). Prediction of short linear protein binding regions. Mol Biol.

[14] Davey NE, Shields DC, Edwards RJ (2006). SLiMDisc: short, linear motif discovery, correcting for common evolutionary descent. Nucleic Acids Res.

[15] Edwards RJ, Davey NE, and Shields DC. SLiMFinder: a probabilistic method for identifying overrepresented, convergently evolved, short linear motifs in proteins. PLos One. 2007;2:e967. doi: 10.1371/journal.pone.0000967. 10.1371/journal.pone.0000967 PMC1989135 17912346

[16] Dosztányi Z, Csizmok V, Tompa P, Simon I (2005). IUPred: web server for the prediction of intrinsically unstructured regions of proteins based on estimated energy content. Bioinformatics.

[17] Altschul SF, Madden TL, Schaffer AA, Zhang JH, Zhang Z, Miller W, Lipman DJ (1997). Gapped blast and psi-blast: a new generation of protein database search programs. Nucleic Acids Res.

[18] Faraggi E, Xue B, Zhou Y. Improving the prediction accuracy of residue solvent accessibility and real-value backbone torsion angles of proteins by fast guided-learning through a two-layer neural network. Proteins. 2009;74:847–56. 10.1002/prot.22193 PMC2635924 18704931

[19] Schlessinger A, Yachdav G, Rost B (2006). PROFbval: predict flexible and rigid residues in proteins. Bioinformatics.

[20] Malhis N, Wong ETC, Nassar R, and Gsponer J. Computational identification of Morfs in protein sequences using hierarchical application of bayes rule. PLoS ONE. 2015;10:e0141603. doi: 10.1371/journal.pone.0141603. 10.1371/journal.pone.0141603 PMC4627796 26517836

[21] Malhis N, Gsponer J (2015). Computational identification of MoRFs in protein sequences. Bioinformatics.

[22] Dubchak l, Muchnik l, and Kim SH. Protein folding class predictor for SCOP: approach based on global descriptors, ISMB-97 Proceedings Int Conf Intell Syst Mil Biol. 1997;5:104–107. 9322023

[23] Sharma A, Paliwal KK, Dehzangi A, Lyons J, Imoto S, Miyano S. A strategy to select suitable physicochemical attributes of amino acids for protein fold recognition. BMC Bioinformatics. 2013;14:233. doi: 10.1186/1471-2105-14-233. 10.1186/1471-2105-14-233 PMC3724710 23879571

[24] Dehzangi A, Paliwal KK, Lyons J, Sharma A, Scattar A. A segmentation-based method to extract structural and evolutionary features for protein fold recognition. IEEE/ACM Trans Comput Biol Bioinform. 2013;11:510–19. 10.1109/TCBB.2013.2296317 26356019

[25] Sharma A, Lyons J, Dehzangi A, Paliwai KK (2013). A feature extraction technique using bi-gram probabilities of position specific scoring matrix for protein fold recognition. Theor Biol.

[26] Lyons J, Dehzangi A, Heffernan R, Yang Y, Zhou Y, Sharma A, Paliwal K (2015). Advancing the accuracy of protein fold recognition by utilizing profiles from hidden Markov models. IEEE Transaction on Nanabioscience.

[27] Sharma A, Sharma R, Dehzangi A, Lyons J, Paliwal K, and Tsunoda T. Importance of dimensionality reduction in protein fold recognition. 2nd Asia-Pacific World Congress on Computer Science and Engineering (APWC on CSE). Nadi Fiji; 2015.

[28] Mousavian Z, Khakabimamaghani S, Kavousi K, Masoudi-Nejad A (2016). Drug–target interaction prediction from PSSM based evolutionary information. J Pharmacol Toxicol Methods.

[29] Lyons J, Paliwal K, Dehzangi K, Hefferman A, TatsuhikoTsunoda R, Sharma A (2016). Protein fold recognition using HMM–HMM alignment and dynamic programming. J Theor Biol.

[30] Remmert M, Biegert A, Hauser A, Söding J (2011). HHblits: lightning-fast iterative protein sequence searching by HMM-HMM alignment. Nat Methods.

[31] Chang CC, Lin CJ (2011). LIBSVM : a library for support vector machines. ACM Trans Intell Syst Technol.

